# Drug-induced hypersensitivity syndrome by liposomal amphotericin-B: a case report

**DOI:** 10.1186/s13104-015-1486-0

**Published:** 2015-09-30

**Authors:** Mao Hagihara, Yuka Yamagishi, Jun Hirai, Yusuke Koizumi, Hideo Kato, Yukihiro Hamada, Katsuhiko Matsuura, Hiroshige Mikamo

**Affiliations:** Department of Infection Control and Prevention, Aichi Medical University School of Medicine, 1-1 Yazakokarimata, Nagakute, Aichi 480-1195 Japan; Department of Pharmacy, Aichi Medical University School of Medicine, Nagakute, Aichi 480-1195 Japan

**Keywords:** Liposomal amphotericin-B, Drug-induced hypersensitivity syndrome (DIHS), Drug rash with eosinophilia and systemic symptoms (DRESS)

## Abstract

**Background:**

Liposomal amphotericin-B (Ambisome^®^) is widely used antifungal drug for treatments of invasive fungal infections. The use of liposomal amphotericin-B is increasing in medical setting because of its tolerability and potent antifungal activity.

**Case presentation:**

In a case of a 76 year-old Japanese female was admitted with subarachnoid hemorrhage, the ethnicity of the patient is Asian, we experienced that liposomal amphotericin-B was the culprit drug for Drug-induced hypersensitivity syndrome, also known as drug rash with eosinophilia and systemic symptoms in view of a clear temporal relationship between liposomal amphotericin-B administration and the onset of symptoms, the remission of the symptomatological pattern after liposomal amphotericin-B withdrawal.

**Conclusion:**

The present case report shows that prolonged liposomal amphotericin-B treatment can be associated with drug rash with eosinophilia and systemic symptoms. We recommend careful monitoring of neutrophil counts in a prolonged treatment course with liposomal amphotericin-B.

## Background

Drug-induced hypersensitivity syndrome (DIHS), also known as drug rash with eosinophilia and systemic symptoms (DRESS), is a severe adverse drug reaction [1–3]. Its true incidence is unknown, but it has been estimated to occur at the frequency of 1 in 1000 to 1 in 10,000 exposures to high-risk drugs [4]. DRESS syndrome characterized by fever, skin rash, and facial edema, organ involvement such as hepatitis or nephritis. Lymphadenopathy and splenomegaly may occur. The syndrome occurs within 2–6 weeks after initiating drug treatment [5].

Liposomal amphotericin-B (L-AMB; Ambisome^®^) is widely used antifungal drug for treatments of invasive fungal infections [6]. This lipid formulation consists of amphotericin-B embedded in the wall of a unilamellar liposome. A regimen of 2.5–5 mg/kg of body weight/day is effective for treatments of invasive infections caused by *Candida* spp. and *Aspergillus* spp. [7, 8]. L-AMB is also used for treatments of *Cryptococcal* meningitis and mucormycosis [9, 10]. Most noteworthy side effects of L-AMB are hypokalemia and renal insufficiency. Other side-effects, definitely attributed to L-AMB therapy, are low back pain, nausea and vomiting, confusion, rise in alkaline phosphatase, and cholecystitis. However, hypersensitivity with rash and pruritus has been described in rare cases [11]. We report here a clinical observation of L-AMB-induced DRESS.

## Case presentation

**Fig. 1:**
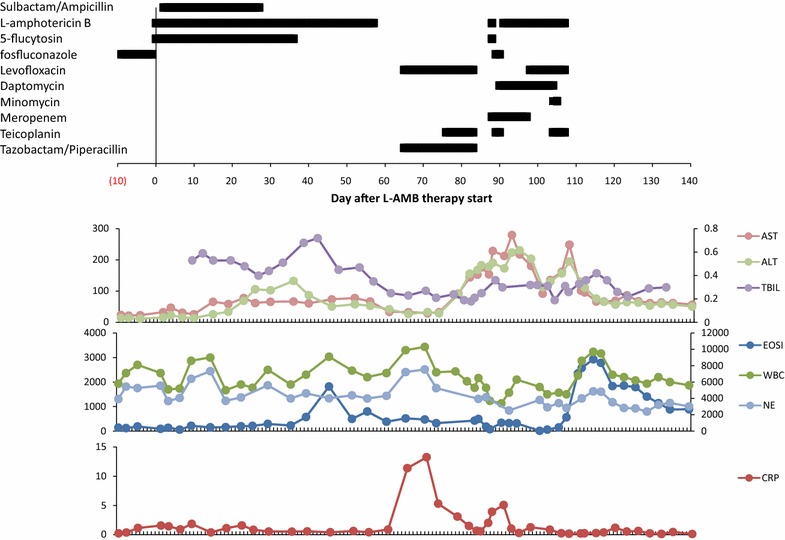
**a** Treatment history and laboratory data. The duration of antibiotic treatment is shown by the *block bars*

A 76 year-old Japanese female with no known drug allergies was admitted with subarachnoid hemorrhage (SAH). The ethnicity of the patient was Asian. Her medical history showed rheumatoid arthritis; anti-inflammatory drug was done with prednisolone (1 mg/day). The persistent high fever and candidemia were admitted after coil embolization for SAH. The patient was prescribed Fosfluconazole (F-FLCZ) at 400 mg/day. One month after the surgery, she had been described as mycotic endophthalmitis with *Candida parapsilosis*. [Minimum inhibitory concentration (MIC) detected by broth microdilution method according to Clinical and Laboratory Standards Institute (CLSI) 94 M27-A3 guideline for several antifungal drugs are as follows; 5-flucytosin (5-FC): ≤0.125 μg/mL, amphotericin-B (AMPH-B): 0.25 μg/mL, fluconazole (FLCZ): 0.125 μg/mL, voriconazole (VRCZ): ≤0.015 μg/mL, micafungin (MCFG): ≤0.03 μg/mL] The summary of antibiotic treatments and laboratory results given in Fig. 1. Because of persistent high fever, candidemia and exacerbation of patient’s clinical condition, the antifungal drug was switched to L-AMB 100 mg/day (3 mg/kg: infusion time was about 2 h) and 5-FC 3000 mg/day. She had been administrated L-AMB and 5-FC for 58 and 37 days. Forty-five days after start of the antifungal combination therapy, the patient was feverish with an exanthema of the trunk, arms and legs, and skin rash appeared. Then, we suspected that 5-FC was the cause drug and 5-FC was ceased. But she had been admitted persistent feverish with an exanthema during L-AMB therapy continued. Her condition has clinically improved with only residual hyper pigmentation after stopped all antibiotics including L-AMB.

**Fig. 2:**
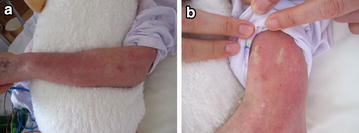
**a**, **b** A macropapular eruption with purpura was present on the upper arm

One month after the event, she had been admitted persistent high fever and re-prescribed L-AMB at 100 mg/day as a prophylactic antifungal drug for candidemia. Right after re-start of the drug therapy, the patient was feverish with an exanthema of the trunk, arms and legs again (Fig. 2). On the physical examination, her temperature was over 38.0 °C and a generalized, diffuse, maculopapular, erythematous, petechial, pruritic rash was noted over the face, trunk, and extremities with marked facial edema, while there was no blister. A maculopapular eruption was noted. The mucosa was not affected, as there was no sore. She also had cervical and inguinal lymphadenopathy. All antimicrobial therapies were stopped as being the most likely cause 20 days after from re-start of L-AMB therapy.

Laboratory examination revealed the following abnormalities: an elevated C-reactive protein (5.08 mg/dL), increased white cell count (6300 cells/mm^3^) and eosinophil (2929 cells/μl), elevated liver enzymes (aspartate aminotransferase 280 U/l; alanine transferase 224 U/l), and decreased estimated glomerular filtration rate; eGFR (75 mg/min). Additionally, as differential diagnosis for fever, rush, virus infection and increment of rheumatism, we investigated some other examinations. Consequently, myeloperoxidase-antineutrophil cytoplasmic antibody (MPO-ANCA) was negative (<1.0 U/ml). Ferritin was 230.7 ng/ml (negative for adult onset Still’s disease). Antibody titres against Human Parvovirus B19 (HPV/B19) IgM was not increased (0.30: upper range is 0.8). Human T-lymphotropic Virus-1 (HTLV-1) was negative. Arthritis and MMR-3 were negative.

A DRESS syndrome was suspected based on the diagnostic criteria for DRESS defined and used by the International Regi-SCAR-group and published by Kardaun et al. [1, 2]. The score was 6, which classified as “possible, probable, or defined”. Additionally, we admitted hypereosinophilia in here laboratory test especially right after second L-AMB therapy started. Her fever went down and the eruption disappeared completely after over 3 weeks from all antibiotic therapy stopped, respectively. We admitted long-lasting skin eruptions in combination with visceral involvement, as one of the typical characters of DRESS. Liver enzyme levels also returned gently to the normal level.

## Discussion

DRESS is a severe, cutaneous reaction to drugs leading to long-lasting skin eruptions in combination with visceral involvement. The hallmark features include a diffuse maculopapular rash, exfoliative dermatitis, facial edema, lymphadenopathy, fever, multivisceral involvement, eosinophilia, and lymphocytosis [12]. In general, one of the most common causative drugs is antiepileptic drug. However, the patient had not taken any antiepileptic drugs previously.

L-AMB is an antifungal drug that inhibits fungal cell wall synthesis, which is mainly used for treatments of *Candida* spp. and *Aspergillus* spp. infections. The use of L-AMB is increasing in medical setting because of its tolerability and potent fungicidal activity [13].

In this case, we admitted some typical symptom of DRESS including eosinophilia, and lymphocytosis, while atypical lymphocytes were not detected. We believe that L-AMB was the culprit drug in view of a clear temporal relationship between L-AMB administration and the onset of symptoms (45 days, typically 2–6 weeks) [14], the remission of the symptomatological pattern after L-AMB withdrawal. Additionally, she had been admitted allergic reactions right after L-AMB re-start. Only L-AMB was re-used when eosinophilia was admitted for this patient, while many other antibiotics were used at the same time. Finally, based on the Naranjo algorithm, it suggested that the systemic reaction was due to L-AMB [15].

As allergic reactions to L-AMB were previously reported [16], some researchers have shown that lipoprotein association of drug compounds can significantly influence not only the pharmacological and pharmacokinetics of the drug, but also the relative toxicity. In pharmacokinetics study of L-AMB, the drug showed higher transferability to the liver, and its half-life of L-AMB concentration in the liver was much longer than that of L-AMB concentration in the blood [11]. Moreover, the L-AMB’s long half-life in the liver is also much longer than other co-administrated drugs. Probably, this is the reason of long-lasting allergic symptoms.

However, our speculation has some limitations. First, while many articles have reported that DRESS might be associated with human herpes virus (HHV-6), Epstein-Barr virus (EBV), cytomegalovirus (CMV) reactivation [4, 17], these reactivation were not conducted. And patch test and the lymphocyte transformation test (LTT) were not investigated in this case. But, the Regi-SCAR-Group Diagnosis Score was 6 [1, 2]. Hence, it is highly possible that this patient could be diagnosed with DRESS. However, long half-life of the drug concentration in tissue of L-AMB and, on the stand points of timing, the drug was highly suspicious.

## Conclusions

The present case report shows that prolonged L-AMB treatment can be associated with DRESS. The use of L-AMB is increasing in medical setting because of its tolerability and potent fungicidal activity. The greater use of L-AMB may result in an increase in the incidence of L-AMB-related adverse effects, while L-AMB is known to have a wide margin of safety [11]. Thus, we recommend careful monitoring of neutrophil counts in a prolonged treatment course with L-AMB.

## Consent

Written informed consent was obtained from the patient for publication of this Case Report and any accompanying images. A copy of the written consent is available for review by the Editor-in-Chief of this journal.

## Abbreviations

L-AMB: Liposomal amphotericin-B
DIHS: Drug-induced hypersensitivity syndrome
DRESS: Drug rash with eosinophilia and systemic symptoms
MIC: Minimum inhibitory concentration
CLSI: Clinical and laboratory standards institute
SAH: Subarachnoid hemorrhage
F-FLCZ: Fosfluconazole
5-FC: 5-flucytosin
MPO-ANCA: Myeloperoxidase-antineutrophil cytoplasmic antibody
HPV/B19: Human parvovirus B19
HTLV-1: Human T-lymphotropic virus-1
EBV: Epstein-barr virus
CMV: Cytomegalovirus
LTT: Lymphocyte transformation test
